# *LRRK2* and *RIPK2* Variants in the *NOD* 2-Mediated Signaling Pathway Are Associated with Susceptibility to *Mycobacterium leprae* in Indian Populations

**DOI:** 10.1371/journal.pone.0073103

**Published:** 2013-08-28

**Authors:** Patrick Marcinek, Aditya Nath Jha, Vidyagouri Shinde, Arun Sundaramoorthy, Raja Rajkumar, Naveen Chandra Suryadevara, Sanjeev Kumar Neela, Hoang van Tong, Vellingiri Balachander, Vijaya Lakshmi Valluri, Kumarasamy Thangaraj, Thirumalaisamy P Velavan

**Affiliations:** 1 Institute of Tropical Medicine, University of Tübingen, Tübingen, Germany; 2 CSIR-Centre for Cellular and Molecular Biology, Hyderabad, India; 3 LEPRA- Blue Peter Public Health and Research Centre, Hyderabad, India; 4 Department of Zoology, School of Life sciences, Bharathiar University, Coimbatore, India; McGill University, Canada

## Abstract

In recent years, genome wide association studies have discovered a large number of gene loci that play a functional role in innate and adaptive immune pathways associated with leprosy susceptibility. The immunological control of intracellular bacteria *M. leprae* is modulated by NOD2-mediated signaling of Th1 responses. In this study, we investigated 211 clinically classified leprosy patients and 230 ethnically matched controls in Indian population by genotyping four variants in *NOD2* (rs9302752A/G), *LRRK2* (rs1873613A/G), *RIPK2* (rs40457A/G and rs42490G/A). The *LRRK2* locus is associated with leprosy outcome. The *LRRK2 rs1873613A* minor allele and respective *rs1873613AA* genotypes were significantly associated with an increased risk whereas the *LRRK2 rs1873613G major* allele and *rs1873613GG* genotypes confer protection in paucibacillary and leprosy patients. The reconstructed *GA* haplotypes from *RIPK2* rs40457A/G and rs42490G/A variants was observed to contribute towards increased risk whereas haplotypes *AA* was observed to confer protective role. Our results indicate that a possible shared mechanisms underlying the development of these two clinical forms of the disease as hypothesized. Our findings confirm and validates the role of gene variants involved in *NOD2-*mediated signalling pathways that play a role in immunological control of intracellular bacteria *M. leprae.*

## Introduction

Leprosy is a chronic infectious disease of the skin and nerves, caused by the bacterium *Mycobacterium leprae*. Despite decreased prevalence in last two decades, the number of new case detection rates remains high as far as in 130 countries, with India contributing to half of the new cases detected worldwide [1]. Host immune responses [2], [3] and genetic factors had been shown to influence the clinical spectrum of leprosy [4]–[7]. Most notable is inter individual variability in disease development, with a wide range of manifestations ranging from lepromatous to tuberculoid leprosy [8]. The lepromatous leprosy is distinguished as borderline lepromatous (BL) and lepromatous (LL) forms and is classified as multibacillary (MB) based on the bacillary load, whereas the tuberculoid leprosy is distinguished as tuberculoid (TT) and borderline tuberculoid (BT) and are classified as paucibacillary (PB) by WHO standards. A difference in immune responses between multibacillary and paucibacillary forms are predicted. In multibacillary or lepromatous forms, the absence of Th1 responses increases the bacilli load with strong humoral immunity [4]. On the other hand, the paucibacillary or the tuberculoid forms reveal an increased Th1 response with limited bacterial load.

The intracellular *M. leprae* has an extended incubation period for up to 30 years. The pathogen driven selection can potentially alter the primed sequence and can direct to substantial changes in gene expression [9]. The pathogen recognition receptors such as *TLRs* (Toll-like receptors) ably recognize the microbes at cell surfaces, whereas PRRs such as nucleotide oligomerization domain (*NOD*) like receptors that are localized in the cytosol can sense and recognize the intracellular pathogens [10]. A recent genome wide study in Chinese leprosy patients has provided vital insights on the role of *NOD2* (rs9302752A/G), *LRRK2* (rs1873613A/G) and *RIPK2* (rs40457A/G and rs42490G/A) variants in regulating the leprosy infection [7]. In addition, the expression of these genes has been shown to up regulate in leprosy in comparison to normal tissues [11]. The Nucleotide-binding oligomerization domain 2 (*NOD2*) located on the long arm of chromosome 16 (16q21) is an intracellular microbial sensor for muramyl dipeptide, a component of bacterial peptidoglycan [12]. Dysregulation in *NOD2* signalling is associated with pathogenesis of many inflammatory disorders [13] and is also associated with triggering of *IL-32* dependent dendritic cell programming in leprosy [14]. The cytosolic pattern recognition receptor *NOD1* and *NOD2* also activates the *RIPK2* gene [15]. The gene encoding receptor- interacting serine-threonine kinase 2 (*RIPK2*) located on the long arm of chromosome 8 (8q21) is essential for signaling through the Toll-like receptors [15], [16]. In addition, the *RIPK2* interaction with NOD2 enhances NF-κB activity making it an important player in cellular immune response [17]. The Leucine-rich repeat serine/threonine-protein kinase 2 (*LRRK2*) variants located on the long arm of chromosome 12 (12q12) are well documented as a common cause for parkinson disease [18]. Also *LRRK2* gene variants were also documented for their role in inflammatory diseases [19] and to microbial infections [20], [21]. During onset of early leprosy infection, *M. leprae* antigens are presented to CD4^+^T cells, which activate the Th1 responses resulting in interferon gamma production leading to macrophages maturation and subsequent killing. *NOD2* and *RIPK2* regulate the interferon- gamma production [7].

Initiation of NOD2 signalling is mediated by *RIPK2* by an ubiqutination process and involvement of TAK1 and nuclear factor-κB essential modulator to the NOD2-RIPK2 complex leads to the movement of NF-κB to the nucleus and subsequent activation of NF-κB target genes [22]. NOD2-mediated signaling pathway plays an essential role in the immunological control of intracellular bacteria. We investigated the possible association of gene variants *NOD2* (rs9302752A/G), *LRRK2* (rs1873613A/G) and *RIPK2* (rs40457A/G and rs42490G/A) that are vital for NOD2 signalling and subsequent activation of the NF-kB complex in a cohort of clinically classified leprosy patients.

## Materials and Methods

### Ethical Statement

Informed written consent was received from all leprosy patients. The study was approved by the research advisory committee and institutional ethical committee of LEPRA- Blue peter public health research centre, Hyderabad, India. Informed written consent was also obtained from all the normal individuals and the institutional ethical committee of CCMB has approved this study.

### Sampling

All the leprosy patients studied (n = 211) were outpatients and were recruited at the LEPRA- Blue Peter Public Health and Research Centre (BPHRC) in Hyderabad, India [23]. Patients were clinically evaluated and graded by the physicians either as a paucibacillary (PB, n = 74) or multibacillary (MB, n = 137) group, based on WHO standards [24]. Based on the number of lesions and presence of acid-fast bacilli (AFB) in skin slit smears taken from at least five different places of the body (both earlobes, both halves of the forehead, at least one from one of the lesions) leprosy patients were classified. More than five lesions as well as presence of acid fast bacilli in any of the smears precludes a multibacillary diagnosis, while a number of less than five lesions and, more importantly, a lack of acid fast bacilli in any of the smears is considered as the paucibacillary presentation. In addition to the patients, blood samples from individual controls (n = 230) were collected from adult males and females (18–35 years) form the same ethnicity.

### Genotyping

DNA was isolated from blood utilizing the DNeasy Blood and Tissue kit (Qiagen, Germany) following the protocol of the manufacturer. The primer sequences utilized for genotyping with primer specific annealing temperatures is summarized in Table 1. In brief: PCR was carried out in a 20 µl reaction volume with 5 ng of genomic DNA, 1× PCR buffer (20 mM Tris-HCl pH 8.4, 50 mM KCl, 1.5 mM of MgCl2; Qiagen), 0.125 mM of dNTPs, 0.5 mM of each primer and 1 U Taq DNA polymerase (Qiagen, Hilden, Germany) on a PTC-200 Thermal cycler (MJ Research, USA). Thermal cycling parameters for amplification were: initial denaturation at 94°C for 5 min, followed by 35 cycles of respectively 15 sec at 94°C for denaturation, 60 sec at primer specific annealing temperature, and 60 sec at 72°C extension. This was followed by a final extension of 10 min at 72°C. PCR products were cleaned up using Exo-SAP-IT (USB, Affymetrix, USA) and 1 µl of the purified product were directly used as templates for sequencing, using the BigDye terminator v. 2.0 cycle sequencing kit (Applied Biosystems, USA) on an ABI 3130 XL and ABI 3730×L DNA sequencer, according to the manufacturer’s instructions. Polymorphisms were identified by assembling the sequences with respective reference sequences obtained from SNPper database (http://snpper.chip.org) using Codon code Aligner 4.0 software (http://www.codoncode.com/) and were reconfirmed visually from their respective electropherograms.

**Table 1:** Investigated SNP variants in leprosy patients and controls.

SNP ID	Locus	Gene	SNP	Primer Pairs (5′–3′)	Tm [°C]
rs9302752	16q21	*NOD2*	A/G	F: GCCTTTGTTTTCGCAGTTCCTTCAG	55
				R: CCTCGGTGACCACTTCTCTGCATTC	
rs1873613	12q21	*LRRK2*	A/G	F: CACCCAAGACACACAAGGAAAAAGCATATA	55
				R: GCCTTCTTACGTTTTACCTCCCCCTCTT	
rs40457	8q21	*RIPK2*	A/G	F: GATTTTCCCCCAGAAGAAGG	50
				R: GCAGGAAAATGAATCCATGA	
rs42490	8q21	*RIPK2*	G/A	F: ACCCACTTCCTCCCTACCACAATCTG	55
				R: GCGGAATAGCTGGATCTCTCACACA	

### Statistical Analysis

Data was analyzed using STATA and the level of significance was set to a p-value of <0.05. The distribution of genotypes between control and leprosy patients, as well as between controls and clinically classified patients were analyzed by two tailed fisher exact tests. Genotype or haplotype frequencies were analyzed by simple gene counting and expectation-maximum (EM) algorithm and the significance of deviations from Hardy-Weinberg equilibrium was tested using the random-permutation procedure as implemented in the Arlequin v. 3.5.1.2 software. (http://lgb.unige.ch/arlequin). The comparison of Linkage disequilibrium (LD) for *RIPK2* variants for each patient group as well as for controls were computed using the Haploview v4.2 software that utilizes a default algorithm and this algorithm ignores markers with minor allele frequencies (MAF) <0.05.

## Results

The observed distribution of genotypes and alleles in both clinically classified patients and controls were summarized in Table 2. The observed genotype and allele frequencies of all studied SNPs in clinically classified patient groups and controls were in Hardy-Weinberg equilibrium (P>0.05) except for the *NOD2* SNP rs9302752 (P<0.05). Therefore the *NOD2* SNP rs9302752 was excluded for further association analysis.

**Table 2:** Distribution of investigated *NOD2*, *RIPK2*, and *LRRK2* variants in clinically classified leprosy patients and controls.

Loci	Patients n = 211(%)	MB (LL+LB) n = 137(%)	LL n = 62 (%)	LB n = 75 (%)	PB (BT+TT) n = 74 (%)	BT n = 70 (%)	TT n = 4 (%)	Controls n = 230 (%)	Patients vs. Controls		MB vs. Controls		PB vs. Controls	
									OR (95% CI)	*P value*	OR (95% CI)	*P value*	OR (95%C I)	*P value*
***NOD2_rs9302752***														
*AA*	120(56.9)	84 (61.3)	52(83.9)	32 (42.7)	36 (48.6)	34 (48.6)	2 (50)	114(49.6)		NA		NA		NA
*AG*	58 (27.5)	32 (23.4)	9 (14.5)	23 (30.7)	26 (35.1)	25 (35.7)	1 (25)	104(45.2)		NA		NA		NA
*GG*	33 (15.6)	21 (15.3)	1 (1.6)	20 (26.6)	12 (16.2)	11 (15.7)	1 (25)	12 (5.2)		NA		NA		NA
*A*	298(70.6)	200(73)	113 (91)	87 (58)	98 (66.2)	93 (66.4)	5 (62.5)	332(72.2)						
*G*	124(29.4)	74 (27)	11 (9)	63 (42)	50 (33.8)	47 (35.6)	3 (37.5)	128(27.8)		NA		NA		NA
***LRRK2_rs1873613***														
*GG*	73 (34.6)	56 (40.9)	27(43.6)	29 (38.7)	17 (23)	16 (22.8)	1 (25)	112(48.7)	0.56 (0.37–0.83)	0.0028		NS	0.31 (0.16–0.6)	0.0001
*AG*	93 (44.1)	62 (45.2)	25(40.3)	37 (49.3)	31 (41.9)	30 (42.9)	1 (25)	91 (39.6)		NS		NS		NS
*AA*	45 (21.3)	19 (13.9)	10(16.1)	9 (12)	26 (35.1)	24 (34.3)	2 (50)	27 (11.7)	2.04 (1.2–3.6)	0.007		NS	4.1 (2.1–7.9)	0.000014
*G*	239(56.6)	174 (63.5)	79(63.7)	95 (63.3)	65 (43.9)	62 (44.3)	3 (37.5)	315(68.5)	0.61 (0.45–0.8)	0.0003		NS	0.36 (0.240.54)	0.00000017
*A*	183(43.4)	100 (36.5)	45(36.3)	55 (36.7)	83 (56.1)	78 (55.7)	5 (62.5)	145(31.5)	1.7 (1.25–2.2)	0.0003		NS	2.77 (1.9–4.1)	0.00000017
***RIPK2_rs40457***														
*AA*	117(54.5)	77 (56.2)	32(51.6)	45 (60)	40 (54)	37 (52.9)	3 (75)	144(62.6)		NS		NS		NS
*AG*	79 (37.4)	50 (36.5)	25(40.3)	25 (33.3)	29 (39.2)	28 (40)	1 (25)	74 (32.2)		NS		NS		NS
*GG*	15 (7.1)	10 (7.3)	5 (8.1)	5 (6.7)	5 (6.8)	5 (7.1)	0	12 (5.2)		NS		NS		NS
*A*	313(74.2)	204 (74.5)	89(71.8)	115(76.7)	109 (73.6)	102(72.9)	7 (87.5)	362(78.7)						
*G*	109(23.8)	70 (25.5)	35(28.2)	35 (23.3)	39 (26.4)	38 (27.1)	1 (12.5)	98 (21.3)		NS		NS		NS
***RIPK2_rs42490***														
*GG*	82 (38.9)	55 (40.1)	23(37.1)	32 (42.7)	27 (36.5)	26 (37.1)	1 (25)	85 (37)		NS		NS		NS
*GA*	90 (42.7)	59 (43.1)	23(37.1)	36 (48)	31 (41.9)	28 (40)	3 (75)	104(45.2)		NS		NS		NS
*AA*	39 (18.4)	23 (16.8)	16(25.8)	7 (9.3)	16 (21.6)	16 (22.9)	0	41 (17.8)		NS		NS		NS
*G*	254(60.2)	169 (61.7)	69(55.6)	100(66.7)	85 (49.2)	80 (57.1)	5 (62.5)	274(59.6)						
*A*	168(39.8)	105 (38.3)	55(44.4)	50 (33.3)	63 (50.8)	60 (42.9)	3 (37.5)	186(40.4)		NS		NS		NS

In *LRRK2* gene locus, we observed that the minor allele *LRRK2 rs1873613A* and homozygous genotype *rs1873613AA* were more frequent in leprosy patients than in controls conferring an increased risk of leprosy (OR = 1.7, 95% CI = 1.25–2.2, P = 0.0003 and OR = 2.04, 95% CI = 1.2–3.6, P = 0.007, respectively). Whereas major allele *LRRK2 rs1873613G* and homozygous genotype *rs1873613GG* were observed less frequently in patients compared to controls conferring a decreased risk of leprosy (OR = 0.61, 95% CI = 0.45–0.8, P = 0.0003 and OR = 0.56, 95% CI = 0.37–0.83, P = 0.0028, respectively). When clinically classified paucibacillary (PB) patients were compared to controls, a similar trend was observed with a stronger significance (for the minor allele *rs1873613A*: OR = 2.77, 95% CI = 1.9–4.1, P<0.0001; for the homozygous genotype *rs1873613AA*: OR = 4.1, 95% CI = 2.1–7.9, P = P<0.0001). These results may confer that the *LRRK2 rs1873613A/G* contributed to the progression of paucibacillary leprosy. However, there was no significant difference of allele and genotype frequencies in comparison to multibacillary (MB) patients with controls (Table 2). In addition, we did not observe any significant difference of heterozygous genotype frequency in all comparisons.

Two loci of *RIPK2* gene (rs40457A/G and rs42490G/A) were investigated in this study. However, no significant difference of allele and genotype frequency of neither SNP rs40457A/G or rs42490G/A was observed in all the comparisons. In leprosy patients, both the studied *RIPK2* variants were observed to be in high LD [Leprosy patients (D′ = 0.64, LOD = 12.72, r^2^ = 0.2), MB patients (D’ = 0.53, LOD = 5.84, r^2^ = 0.56), PB patients (D′ = 0.84, LOD = 7.58, r^2^ = 0.34)], whereas in controls it was observed in a low degree of linkage disequilibrium (D′ = 0.38, LOD = 3.2, r^2^ = 0.06)]. We reconstructed haplotype based on these two studied *RIPK2* SNPs (rs40457A/G and rs42490G/A). Four haplotypes and their frequencies were observed including *AG*, *AA*, *GA* and *GG*. The *RIPK2* haplotype *GA* was observed more frequently in leprosy patients compared to controls inferring an increased risk of leprosy (OR = 1.46, 95% CI = 1.02–2.1, P = 0.036), whereas *RIPK2* haplotype *AA* was observed less frequently in leprosy patients compared to controls inferring a protection against leprosy (OR = 0.69, 95% CI = 0.49–0.97, P = 0.028). In addition, *RIPK2* haplotype *GA* was observed more frequently in paucibacillary (PB) patients in comparison to controls inferring an increased risk of leprosy (OR = 1.8, 95% CI = 1.1–2.8, P = 0.018). Furthermore, there was no significant difference of *RIPK2* haplotype frequencies in comparison between multibacillary (MB) patients with controls (Table 3).

**Table 3:** Distribution of investigated RIPK2 haplotypes in clinically classified leprosy patients and controls.

*RIPK2* haplotype(rs40457/rs42490)	Patientsn = 422(%)	MB (LL+LB)n = 274(%)	LLn = 124(%)	LBn = 150(%)	PB (BT+TT)n = 148(%)	BTn = 140(%)	TTn = 8(%)	Controls n = 460 (%)	Patients vs. Controls		MB vs. Controls		PB vs. Controls	
									OR (95% CI)	*P value*	OR (95% CI)	*P value*	OR (95% CI)	*P value*
*AG*	234 (55.5)	152 (55.5)	66 (53.2)	86 (57.3)	82 (55.4)	77 (55.0)	5 (62.5)	247 (53.7)		NS		NS		NS
*AA*	79 (18.7)	52 (19.0)	23 (18.6)	29 (19.3)	27 (18.3)	25 (17.9)	2 (25.0)	115 (25.0)	0.69 (0.49–0.97)	0.028		NS		NS
*GA*	89 (21.1)	53 (19.3)	32 (25.8)	21 (14.0)	36 (24.3)	35 (25.0)	1 (12.5)	71 (15.4)	1.46 (1.02–2.1)	0.036		NS	1.8 (1.1–2.8)	0.018
*GG*	20 (4.7)	17 (6.2)	3 (2.4)	14 (9.4)	3 (2.0)	3 (2.1)	0	27 (5.9)		NS		NS		NS

## Discussion

All the four studied innate immune gene variants were hypothesized to play a significant role in controlling the interferon-gamma production and considered as vital modulators for NF-κB [7]. We investigated the role of gene variants in *NOD2* (rs9302752A/G), *LRRK2* (rs1873613A/G) and *RIPK2* (rs40457A/G and rs42490G/A) based on a recently published genome-wide association study (GWAS) that utilized Han Chinese population [7]. Although the studied sample size was lower, the frequencies of all four studied variants were in accordance with Han Chinese population and also in a Gujarati Indian population as reported in HapMap database. The minor allele of *NOD2* rs9302752A/G variant was observed at a similar frequency (0.28) in the studied Indian population to reported frequencies in Han Chinese population (0.22). However, the *NOD2* rs9302752A/G variant was not in Hardy-Weinberg equilibrium in Indian clinically classified leprosy patients and marginally significant in control group. The major allele of *LRRK2* rs1873613A/G variant was also observed at a similar frequency (0.68) compared to reported frequencies in Gujarati Indians in Houston, Texas (GIH) (0.61) as reported in the NCBI Hapmap database. The minor allele of studied *RIPK2* variant rs40457A/G was observed at a similar frequency (0.21) when compared to reported frequencies in Han Chinese (0.25) and in Gujarati Indians in Houston, Texas (GIH) (0.30), whereas the minor allele of *RIPK2* rs42490A/G was observed at a similar frequency (0.41) when compared to reported frequencies in Han Chinese (0.45) and in Gujarati Indians in Houston, Texas (GIH) (0.40).

*NOD2* is an intracellular microbial sensor of the innate immune system that can act as a potent activator and regulator of inflammation in mycobacterial infections [10], [25]. Mutations in the gene encoding *NOD2* in humans have been associated with Crohn’s disease (CD) [26], Blau syndrome (BS) [27], and early onset sarcoidosis (EOS) [28]. In addition, the studied *NOD2* variant (rs9302752A/G) was associated with susceptibility to tuberculosis and leprosy in Chinese and Vietnamese population [7], [29], [30]. NOD2 signaling pathways are activated by a CARD effector domain that causes inflammation by the activation of NF-kB and MAP kinase pathways [10]. Studies have documented the fact that stimulation of NOD proteins are associated with enhanced pro inflammatory cytokine production to *M. leprae* infections [31] and this is well achieved by the recognition of conserved microbial domains by PRRs. Therefore, any alteration in the gene function of PRR domain may possibly reflect towards leprosy susceptibility. Also it was shown that phenotypes of mouse deficient with *NOD2* and *RIPK2* revealed a failure to produce inflammatory cytokines to initiate Th1 responses [32]. In contrast to Han Chinese population [7] we observed that *NOD2* rs9302752A/G variant was not in Hardy-Weinberg equilibrium in studied Indian population, therefore the contribution of this variant to leprosy susceptibility remains unclear. The possibilities for the studied NOD2 variant not in HWE can be due to the fact that Indian populations have been following strict endogamy marriage practices for last tens of thousands years, hence every population acquired unique set of genetic variations. In this study, the leprosy patients represent different ethnic groups from south India and the number from each group is very less. Nevertheless in this study, we always employed ethnically matched control individuals from the same population to avoid the role of population stratification in allelic difference between cases and controls.

For the investigated *LRRK2* rs1873613A/G variant the contribution was observed of this particular variant in the studied Indian population. The minor allele *A* and homozygous genotype *AA* contributed towards an increased risk of leprosy whereas the major allele *G* and homozygous genotype *GG* predisposed as protective factors for leprosy. Our results also showed that *LRRK2* rs1873613A/G variant significantly contributed to the development of paucibacillary leprosy but not multibacillary leprosy. Earlier studies have demonstrated that a trend towards an association between the variant and susceptibility to leprosy [7]. The association study of *LRRK2* rs1873613A/G variant with leprosy was also conducted in a Vietnamese population, however the association was not statistically significant [30]. *LRRK2* is associated with various diseases, including Parkinson’s disease, cancer, and leprosy [33] and also known to be associated with susceptibility to the chronic autoimmune Crohn’s disease, which is an inflammatory disorder [34]. Higher expression of LRRK2 is observed in macrophages and monocytes revealing its significance in the innate immune system [35] and in leprosy per se. Most of the replication studies do not document similar effects of a particular gene variant contribution when investigated in different ethnicities [36]–[38]. In the reported GWAS study from Han Chinese population, the *LRRK2 rs1873613A* allele was observed as a major allele whereas the *rs1873613G* variant is a minor allele and confers decreased risk towards leprosy in Chinese patients. [7]. Additionally similar allele frequencies were reported in the Vietnamese population [30]. However in the studied Indian population, the *LRRK2 rs1873613G* allele was observed as a major allele whereas the *rs1873613A* variant increased the risk towards leprosy in the investigated Indian cohort. There seems a switch of allele frequencies between populations and the clinical significance of the studied variant likely depends on the context of the studied ethnicities. Additionally India is inhabited by the very first out-of-Africa modern human about 65,000 years ago. Since then they remain unmixed, therefore the genome of Indian populations are unique and the allele frequency differ significantly when compare to the rest of the world [39]. *LRRK2* gene has been shown to play an important role in different diseases including Parkinson, Crohn’s and inflammatory diseases [18], [19], [21], [33]. Interestingly, LRRK2 has been also demonstrated to be an IFN-gamma target gene, involve in different immune response signaling such as NF-kB pathways, and contribute to the antibacterial activity of the macrophages, in which LRRK2 plays a role in the killing of intracellular bacteria such as *S. typhimurium* [20]. In addition, the expression of *LRRK2* gene was significantly higher in leprosy compared to normal tissues [11]. This suggested that LRRK2 might contribute crucially to the immune response against intracellular bacteria *M. leprae*.

The *RIPK2* rs40457A/G and rs42490G/A variants did not confer any significance results at allele and genotype levels. A larger sample size will be required in order to detect any significance for the both studied *RIPK2* variant at allele level. We observed the reconstructed haplotypes *AA* and *GA* based on two variants rs40457A/G and rs42490G/A to be significantly associated with leprosy susceptibility. In addition, the haplotype *GA* also conferred to an increased risk of paucibacillary leprosy development. Recently, a replicate study conducted in a Vietnamese population showed that the variant *RIPK2* rs42490G/A was significantly associated with leprosy whereas the significance was not revealed for the variant *RIPK2* rs40457A/G [30].Studies have established the fact that RIPK2 interaction with NOD2 enhances NF-κB activity making it an important player in cellular immune response [17]. A recent study has also documented on the interaction between the *NOD2* and *RIPK2* loci (NOD2-RIPK2 complex) in activating the NF-κB pathway as a part of the host defence response to leprosy infection [40]. Therefore, our results indicate that a possible shared mechanisms as a basis for the development of these two clinical forms of the disease as hypothesized earlier [7].

In conclusion, our study validated the association of gene variants involved in intracellular sensing that are believed to play a role in immunologic control of intracellular bacteria *M. leprae* in Indian leprosy patients. Overall the study increases our understanding on complex molecular and cellular mechanisms that are regulated by the intracellular pathogen *M. leprae* during its clinical course.
